# Epidermal Neuromedin U Attenuates IgE-Mediated Allergic Skin Inflammation

**DOI:** 10.1371/journal.pone.0160122

**Published:** 2016-07-27

**Authors:** Yoshiko Mizukawa, Takaaki Doi, Yoshimi Yamazaki, Akihiko Kudo, Tetsuo Shiohara

**Affiliations:** 1 Department of Dermatology, Kyorin University School of Medicine, Mitaka, Tokyo, Japan; 2 Department of Anatomy, Kyorin University School of Medicine, Mitaka, Tokyo, Japan; French National Centre for Scientific Research, FRANCE

## Abstract

Although keratinocyte-derived neuropeptide neuromedin U (NMU) mediates the proinflammatory effects of innate-type mast cell activation, no information is available on the physiological roles. Here, to investigate the effects of NMU on IgE-mediated allergic skin inflammation, we determined whether IgE-mediated inflammation associated with severe scratching was induced in *Nmu*^*-/-*^ mice administered repeated hapten applications to the ear or footpad. Dry skin was induced by targeted deletion of *Nmu*. Mice administered repeated hapten application developed IgE-mediated allergic inflammation characterized by severe scratching and increased serum IgE levels only when the ear, and not the footpad, was subjected to scratching, indicating that depletion of NMU from the epidermis alone does not drive such allergic inflammation. Thus, the susceptibility of *Nmu*^*-/-*^ mice to allergic inflammation depends primarily on scratching dry skin. Further, allergic skin inflammation mediated by FcεRI cross-linking in *Nmu*^*-/-*^mice was inhibited by prior injection of NMU. These results indicate that NMU plays an important physiological role as a negative regulator during the late stage of IgE-mediated allergic skin inflammation.

## Introduction

Approximately 10% of patients with atopic dermatitis (AD) harbor a loss-of-function mutation in the gene (*Flg*) that encodes filaggrin, which is important for skin barrier function [1]. Patients with AD experience a type of dry skin which induces intense pruritus, causing patients to scratch and injure the skin [2, 3]. While recent research on the pathogenesis of AD has focused on the genetic basis of these skin conditions [4–6], the dry skin induced by defects in these genes such as *CLDN1* does not always result in intense pruritus and scratching. Moreover, previous studies do not provide an answer to the important question of whether a targeted deletion of a single gene could render otherwise refractory animals susceptible to allergic skin inflammation, as observed in AD.

To identify the mechanisms that drive the pathogenesis of AD, it is important to consider the cells that mediate the allergic response. For example, activation of mast cells represents a mechanistic bridge between innate and acquired immunity [7–9] which enables the host to respond to different stimuli by rapidly releasing immune mediators from keratinocytes and modulating mast-cell and T-cell function [7–11]. The neuropeptide neuromedin U (NMU), which is stored in epidermal keratinocytes, is released in response to a variety of stimuli [12, 13], and is therefore likely the leading candidate involved in the control of allergic skin inflammation. Although NMU plays a variety of immunological roles such as in mast cell activation [13], IL-6 production [14], and eosinophil activation [15], its effect in the IgE-mediated allergic skin response is unknown. Here, to determine the role of NMU in the IgE-mediated allergic response, we used a mouse model of allergic skin inflammation induced by repeated hapten application to the ears of Th2-biased mice [16–20]. Th2-biased mice, such as BALB/c mice, are characterized by severe scratching behavior, Th-2-dominated immune responses, and increases in serum antigen-specific IgE that can be induced by repeated application of hapten. They therefore serve as a model for human AD, particularly for extrinsic allergen-driven AD [16–20].

Here, we show that a targeted deletion of *Nmu* caused a profound decrease in skin surface hydration (SSH) in otherwise resistant C57BL/6 (B6) mice. These *Nmu*^-/-^ mice developed IgE-mediated allergic inflammation characterized by severe scratching, increased serum IgE levels, and Th2-dominated responses, despite their original B6 background [17–21]. These observations suggest that epidermal NMU plays an important physiological role in inhibiting IgE-mediated allergic skin inflammation.

## Materials and Methods

### Mice

B6 mice were purchased from Charles River Laboratory Japan (Yokohama, Japan). NMU-deficient mice were generated as reported previously and backcrossed for six generations onto a B6 background [12]. Female mice aged 6 to 10 weeks were used for all experiments. There were no visible skin lesions in B6 or *Nmu*^*-/-*^ mice at 4, 8, 20, or 50 weeks of age. All mice were kept in a specific pathogen-free barrier facility at the Kyorin University animal facility under a controlled room temperature (23–25°C) and humidity (40–50%). All procedures were performed in accordance with the Guidelines of the Animal Care and Use Committee of Kyorin University. To sample tissues, mice were anesthetized with C_3_H_2_CIF_5_O. The Committee on the Ethics of Animal Experiments of Kyorin University approved the protocol (Permit Number: 23–7).

### Reagents

2, 4, 6-Trichlorobenzene (TNCB) was obtained from Tokyo Kasei Co. (Tokyo, Japan) and used as a 1% solution in acetone for sensitization and elicitation, as described previously [17–20]. A polyclonal rabbit anti-NMU serum against the [Cys17]-mouse NMU peptide was a gift from M. Kojima [22], and a monoclonal antibody (mAb) against rabbit IgG conjugated to Alexa Fluor 488 (A-11034) was purchased from Invitrogen (Carlsbad, CA, USA).

### Murine model of chronic allergic inflammation

Mice were sensitized using a single epicutaneous application of 20 μl of 1% TNCB to the left ear or footpad on day –7 followed by repeated application of 1% TNCB or vehicle (1% acetone) to the right ear or footpad from days 0 to days 21–26 at 2-day intervals [17–20]. Tissue thickness (μm) of the ear or footpad was measured using a dial thickness gauge (G-1A, Ozaki, Tokyo, Japan). Hapten-induced ear swelling was measured as tissue thickness before and 0.5 h after elicitation.

### Measurement of scratching behavior

The experimental protocols included minor modifications of published methods [23, 24]. Three days before the experiments, a small magnet was inserted into the dorsum of the right hind paw of an anesthetized mouse. One day before the experiments, mice were placed individually in clear acrylic cages. Scratching was monitored using a MicroAct (Neuroscience, Tokyo, Japan) before and immediately after the initial application of hapten, and for 24 h after each subsequent elicitation. One scratching episode was defined as continuous scratching behind the ear for >0.17 s. No significant difference was noted in the pattern of scratching behavior among 1-h periods from 7:00 AM–7:00 PM or from 7:00 PM–7:00 AM; or during the 24 h period after each elicitation (data not shown). Therefore, the mean number of scratching episodes was determined between 7:00 PM–7:00 AM. To detect baseline levels of spontaneous scratching behavior without the effect of hapten application, scratching behavior on day -9 was also counted.

### Measurement of skin surface hydration and transepidermal water loss

SSH was measured using a Skicon-200 (IBS Co, Hamamatsu, Japan) as described previously [25, 26]. This device evaluates SSH by measuring the conductivity of the skin surface when the probe is applied to the skin [25]. Transepidermal water loss (TEWL) was measured using a Vapometer (Delfin Technologies, Kupio, Finland). All measurements were performed at constant temperature and humidity. Mice were allowed to acclimate to these conditions for at least 30 min before measurements.

### Measurement of serum IgE levels

Blood samples were collected from the tail vein at various times and the serum was stored in a microtube at –80°C. Serum levels of IgE were measured using a commercially available enzyme-liked immunosorbent assay kit (OptEIA, BD Pharmingen, San Diego, CA). All assays were performed according to the manufacturer’s instructions.

### Immunohistochemistry

The experimental protocols for the detection of NMU included minor modifications of methods described previously [13, 22]. To detect NMU, mouse ears were immersed in 4% paraformaldehyde overnight, 6-mm thick sections were prepared, and the sections were reacted with a rabbit anti-NMU serum (gift from M. Kojima, diluted to 1:1000) overnight at 4°C and then with Alexa Fluor 488-conjugated anti-rabbit IgG secondary antibody (diluted to 1:200) (A-11034; Invitrogen, Carlsbad, USA). Sections incubated with an isotype-control rabbit serum instead of the primary mAb served as a negative control. Hematoxylin-eosin and toluidine blue staining were performed as described previously [17–19]. The number of mast cells in ear-tissue sections stained with toluidine blue was determined using an Olympus microscope (40× objective, 10× eyepiece) by two independent observers who were uninformed of the identities of the samples. The assessment of each observer did not differ significantly.

### RT-PCR analysis of cytokine mRNA levels

Forty-eight hours after the final day of the 21-day treatment with TNCB, skin samples were removed from the ears of B6 and *Nmu*^-/-^ mice. Total RNA was extracted from the ear using an RNeasy Fibrous Tissue Mini Kit (Qiagen, Tokyo, Japan). RNA samples served as templates to synthesize cDNA using a High Capacity cDNA Reverse Transcription Kit (Applied Biosystems, CA, USA). Cytokine mRNA levels were measured using RT-PCR with a TaqMan Gene Expression Assay Kit and an ABI PRISM 7700 Sequence Detection System (Applied Biosystems). TaqMan Gene Expression Assay IDs (Applied Biosystems) were as follows: interleukin (IL)-4, Mm9999154_ml; IL-13, Mm9999190_ml; interferon (IFN)-γ, Mm 9999071_ml; IL-17A, Mm00439619_ml; IL-10, Mm9999062_ml; IL-23A, Mm00518984_ml; and glyceraldehyde-3-phosphate dehydrogenase (GAPDH), Mm03302249_gl. PCR conditions were 50°C for 2 min, 95°C for 10 min, and 50 cycles of amplification (95°C for 15 s and 60°C for 1 min). The level of each cytokine mRNA was normalized to that of a control B6 or *Nmu*^-/-^ mouse, respectively

### Flow cytometric analysis of cytokine production in the lymph node draining the ear

Single-cell suspensions were prepared from the draining lymph node (LN) collected from B6 and *Nmu*^*-/-*^ mice on day 23 after repeated application of hapten to the ear. Production of intracellular IL-17 (eBioscience, CA, USA) by single cells was determined using flow cytometry after *in vitro* stimulation of cells with phorbol 12-myristate 13-acetate (Sigma-Aldrich, St. Louis, MO, USA) and ionomycin (Sigma-Aldrich) for 4 h as described previously [27].

### Effect of neuromedin U on immediate-type hypersensitivity (ITH) mediated by FcεRI cross-linking

The right footpads of B6 and *Nmu*^-/-^ mice were initially sensitized with various concentrations of mouse tetranitrophenyl (TNP) hapten-specific IgE (557080; BD Pharmingen) 3 days before initial application of TNCB (S1A Fig). TNCB was applied to the sensitized footpad to induce antigen-specific IgE-mediated ITH responses at 0.5 h. Another type of FcεRI cross-linking was induced by administering a TNP-specific IgE followed by injection of 25 ng of anti-mouse IgE (553413; BD Pharmingen) (S1B Fig). To demonstrate directly the inhibitory effect of NMU on IgE/anti-IgE-mediated ITH responses, 50 pmol of NMU (NMU-23; Peptide Institute, Inc., Osaka, Japan) was injected into the footpads of *Nmu*^*-/-*^ mice one day before administration of anti-mouse IgE. In this experiment, vehicle (PBS) was injected as a control (S1B Fig).

### Statistical analysis

Data are expressed as the mean ± standard error of the mean (SEM). Statistical significance of differences between groups was analyzed using the Mann–Whitney test, the Student *t* test, or Dunnett’s test. *P <* 0.05 indicates a statistically significant difference.

## Results

### Depletion of neuromedin U from the epidermis decreases skin surface hydration

**Fig 1:**
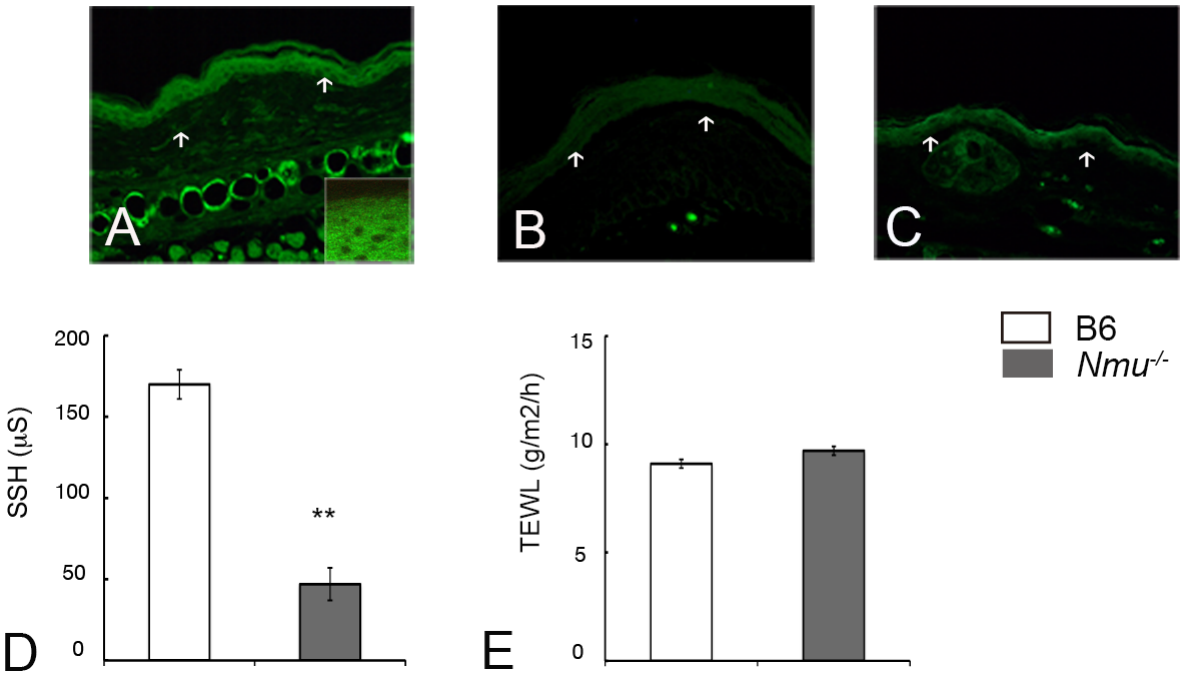
Decrease in SSH associated with depletion of NMU from the epidermis. (A) NMU expression in epidermal keratinocytes in the ears of B6 mice including high magnification (n = 4). (B) NMU expression was not detected in epidermal keratinocytes of the ears of these mice with an isotype control (n = 4). The dermo-epidermal border is indicated by arrows. (C) NMU expression was not detected in the ears of *Nmu*^-/-^ mice. (D) SSH in the ears of B6 and *Nmu*^-/-^ mice (n = 10). (E) TEWL (n = 10). Original magnification, 200×. High magnification, 400×. Data are expressed as the mean ± SEM (n = 10). **P < 0.01.

We first asked whether the absence of NMU in the epidermis was associated with a barrier abnormality that induces downstream immunological abnormalities. To test this possibility, skin-surface impedance analysis was performed to determine the hydration of the ear before treatment. NMU expression was detected in epidermal keratinocytes of the ears of B6 (n = 4, Fig 1A) but not *Nmu*^-/-^ mice, as has been reported previously [13] (n = 4, Fig 1C). Mean SSH of the ears of *Nmu*^-/-^ mice was significantly lower than that of B6 mice (n = 10, Fig 1D). In contrast, TEWL values of the mouse strains did not differ significantly (n = 10, Fig 1E), indicating the absence of any major defect in the epidermal barrier function in *Nmu*^-/-^ mice. These results indicate that the targeted deletion of *Nmu* is associated with decreased SSH in the ear but not with defective epidermal barrier function. We therefore concluded that *Nmu*^-/-^ mice represent a suitable model for determining the role of dry skin in scratching behavior and the subsequent induction of IgE-mediated inflammation.

### Development of IgE-mediated immediate-type hypersensitivity in *Nmu*^-/-^ mice

**Fig 2:**
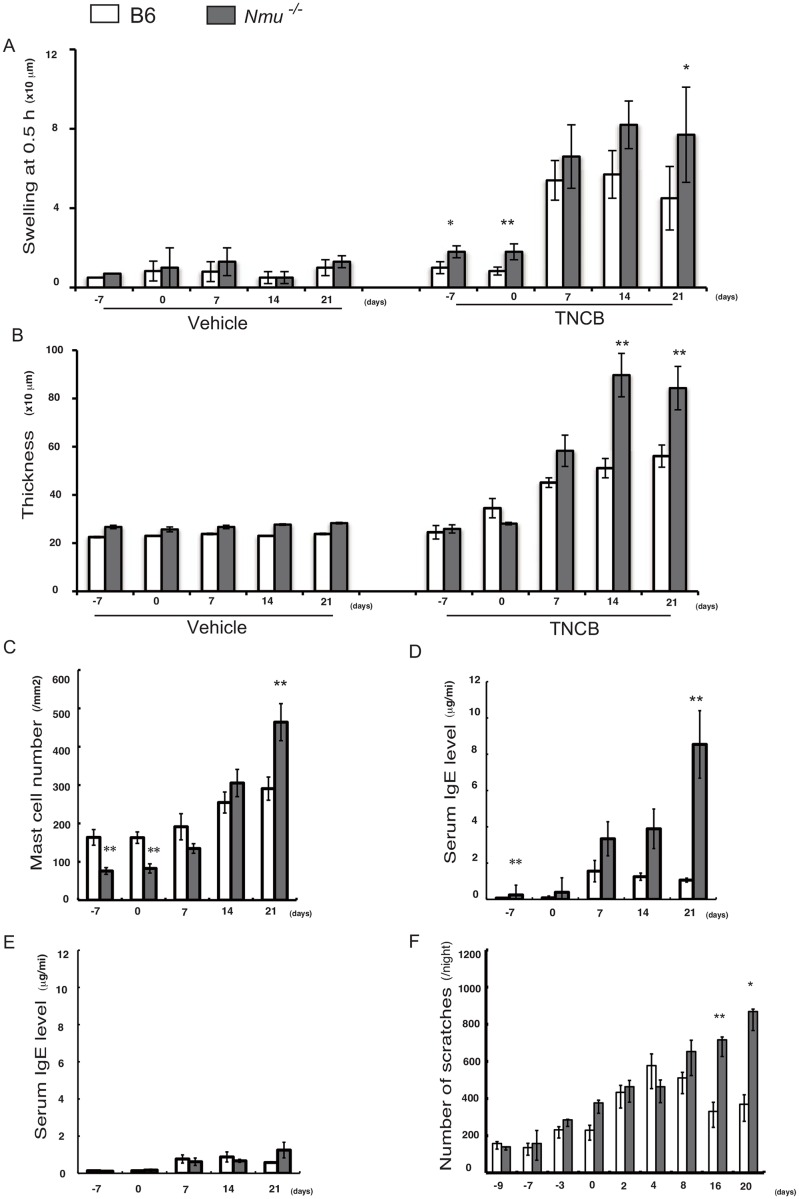
Differences in the responses of mice after repeated hapten application to the ear or footpad. (A) A hapten-specific ITH reaction is indicated by rapid ear swelling 0.5 h after each elicitation (n = 10). (B) Ear thickness (n = 10). (C) Number of mast cells in the ear (n = 5). (D) Serum IgE levels of mice after repeated hapten application to the ear (n = 10). (E) Serum IgE levels of mice after repeated hapten application to the footpad (n = 10). (F) Total number of scratching episodes from 7:00 PM–7:00 AM (n = 10). (A–F) Data are expressed as the mean ± SEM. *P < 0.05, ** *P* < 0.01.

We next asked whether the increased chronic allergic inflammation characterized by itching and scratching that occurs in AD was induced by repeated application of hapten to the ears of *Nmu*^-/-^ mice, as is observed in BALB/c mice. Hapten-specific ITH, ear thickness, number of dermal mast cells, and serum IgE level were significantly increased in *Nmu*^-/-^ mice compared with wild-type B6 mice on day 21 (n = 5–10) (Fig 2A–2D). In contrast, repeated hapten application to the footpad, which is difficult for mice to reach and scratch (n = 10) (Fig 2E), did not induce a significant increase in serum IgE level compared with *Nmu*^-/-^ mice whose ears were repeatedly treated with hapten (n = 10) (Fig 2D).

The mean number of scratches after each application of hapten was greater in *Nmu*^-/-^ compared with B6 mice except on day 4. In particular, a marked increase in the number of scratches in *Nmu*^-/-^ mice was observed on days 16–20 (*P* < 0.01) (n = 10) (Fig 2F). These results indicate that hapten application in the absence of NMU, which is associated with dry skin, evoked scratching behavior that increased serum IgE levels and subsequently induced IgE-mediated inflammation. Thus, scratching associated with repeated hapten application to dry skin in the absence of NMU would render otherwise refractory B6 mice susceptible to IgE-mediated inflammation.

### Histological analysis of skin lesions and cytokine mRNA expression

**Fig 3:**
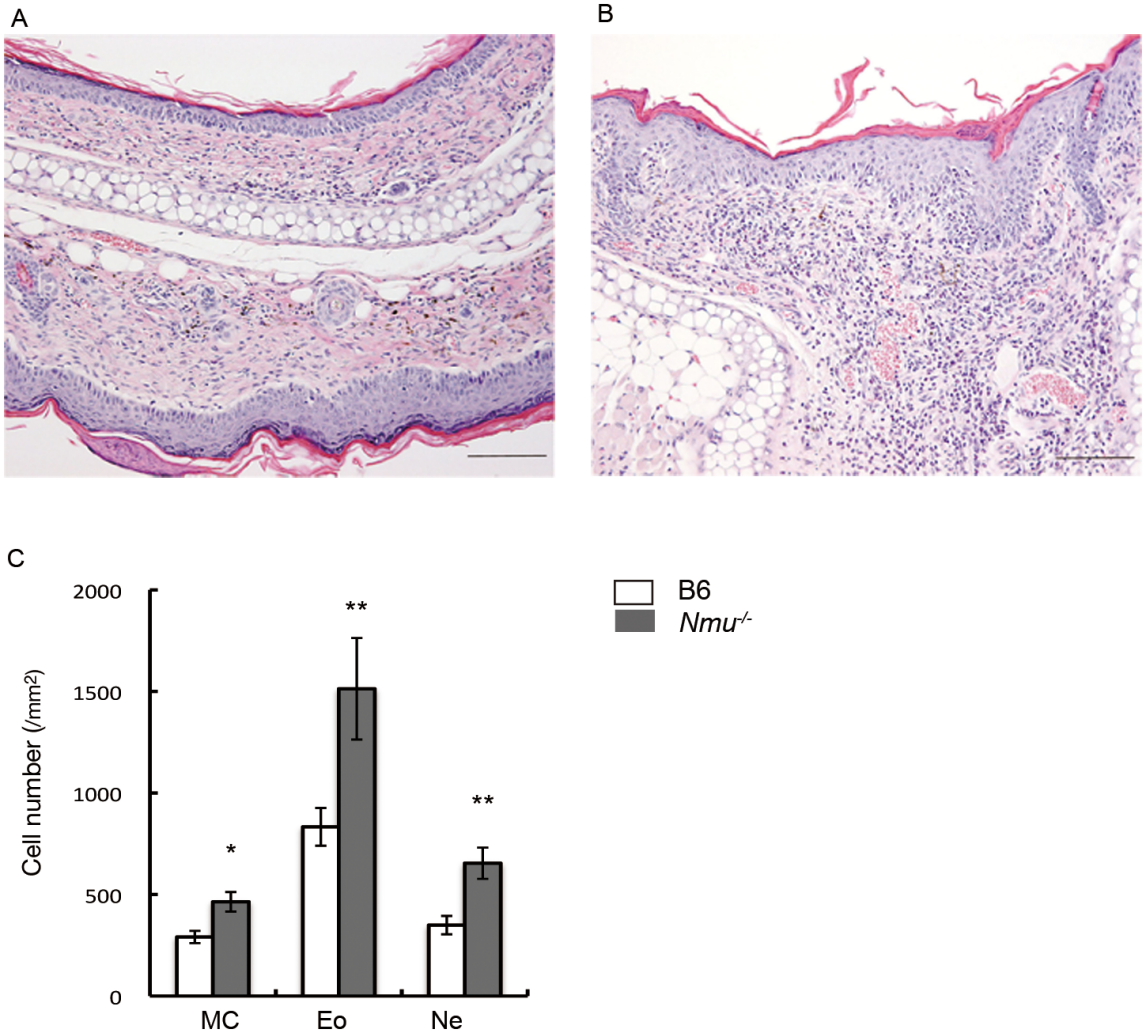
Histological analyses of the ears of B6 and *Nmu*^-/-^ mice after repeated hapten exposure. (A) Hematoxylin-eosin (HE) staining of B6 and (B) *Nmu*^-/-^ mice on day 28 (n = 5). Original magnification, ×100. (C), Numbers of histologically identifiable dermal mast cells (MC), eosinophils (Eo), and neutrophils (Ne) in the ears of B6 and *Nmu*^-/-^ mice (n = 5). *P < 0.05, **P < 0.01.

We observed marked thickening and inflammation in the epidermis and dermis at the site of hapten application to *Nmu*^-/-^ mice. These changes were less apparent in B6 mice (n = 5, Fig 3A and 3B). Further, the numbers of mast cells, eosinophils, and neutrophils increased significantly in *Nmu*^-/-^ mice compared with B6 mice (n = 5, Fig 3C).

**Fig 4:**
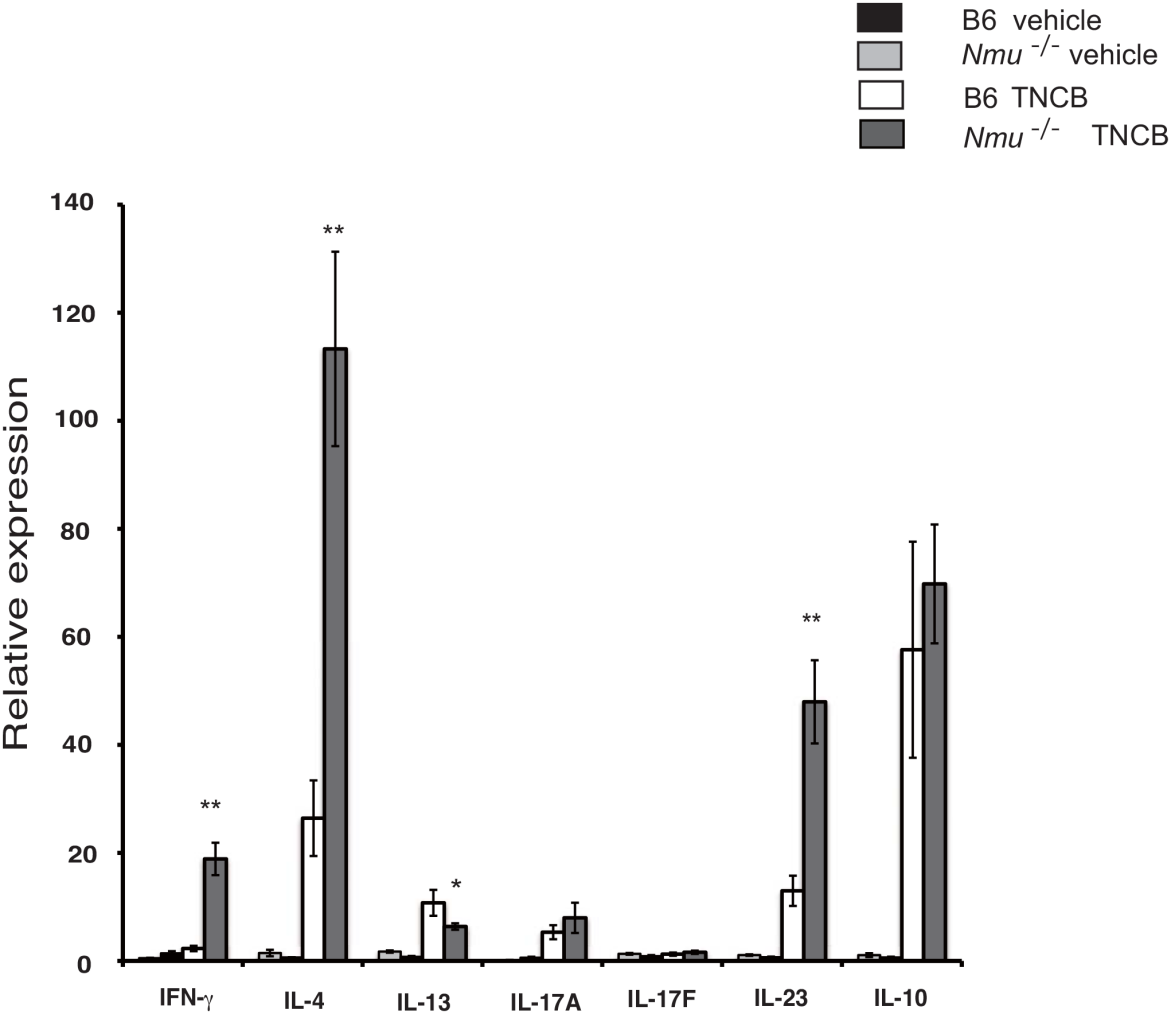
Cytokine mRNA expression in the ears of B6 and *Nmu*^-/-^ mice after repeated hapten application. Results are expressed as fold-increase in expression in the ears of each mouse before hapten application, as assessed by the value of the mean ± SEM (n = 4–6). *P* < 0.05, * *P* < 0.01.

**Fig 5:**
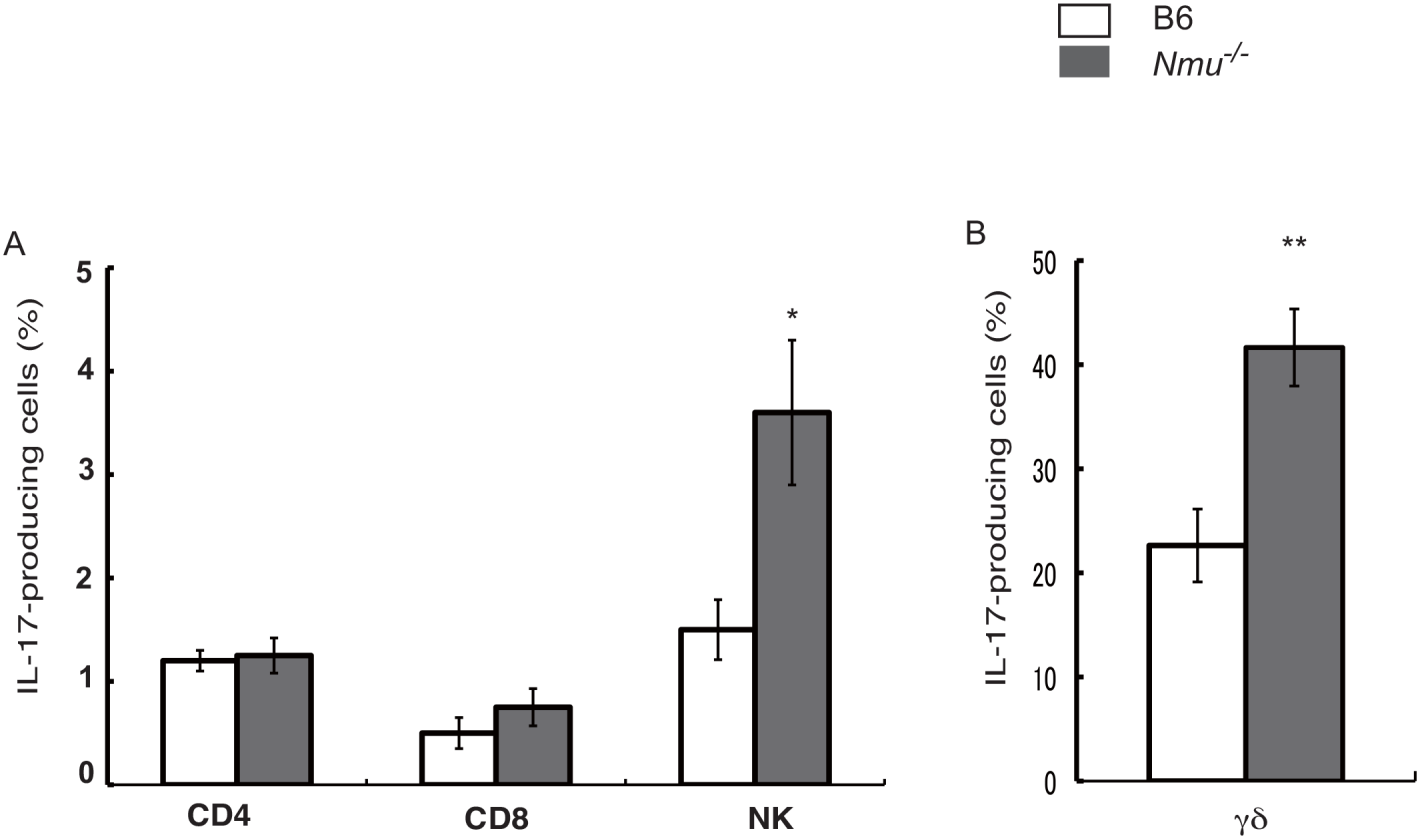
Frequencies of IL-17-producing cells in draining lymph nodes 23 days after repeated hapten application. Mean frequencies of IL-17-producing CD4^+^, CD8^+^, NK (A) and γδ^+^ T cells (B) from B6 (n = 3) and *Nmu*^-/-^ mice (n = 5). Data are expressed as the mean ± SEM.*P < 0.05, **P < 0.01.

We next asked whether repeated application of hapten to the right ears of *Nmu*^-/-^ mice induced a predominant Th2/Th17 response, which typically occurs in skin lesions of humans with AD [28]. RT-PCR analysis revealed markedly increased mRNA levels of IL-4, IFN-γ, and the IL-17-promoting cytokine IL-23 in the skin lesions of *Nmu*^-/-^ mice compared with those in B6 skin (n = 4–6, Fig 4). The levels of IL-17A and IL-17F mRNAs were not significantly increased in *Nmu*^-/-^ mice compared with B6 mice. Further, when we used flow cytometry to determine intracellular cytokine expression in the draining lymph node cells, we detected higher frequencies of IL-17-producing NK and γδ^+^ T cells in *Nmu*^-/-^ mice (n = 5) than in B6 mice (n = 3, Fig 5). These results indicate that the levels of Th2 cytokines as well as those of Th1 cytokines and the IL-17-promoting cytokine IL-23 were upregulated in the skin lesions of *Nmu*^-/-^ mice, as is frequently observed in skin lesions of patients with chronic AD [28].

### Neuromedin U inhibits the development of IgE-mediated immediate-type hypersensitivity

**Fig 6:**
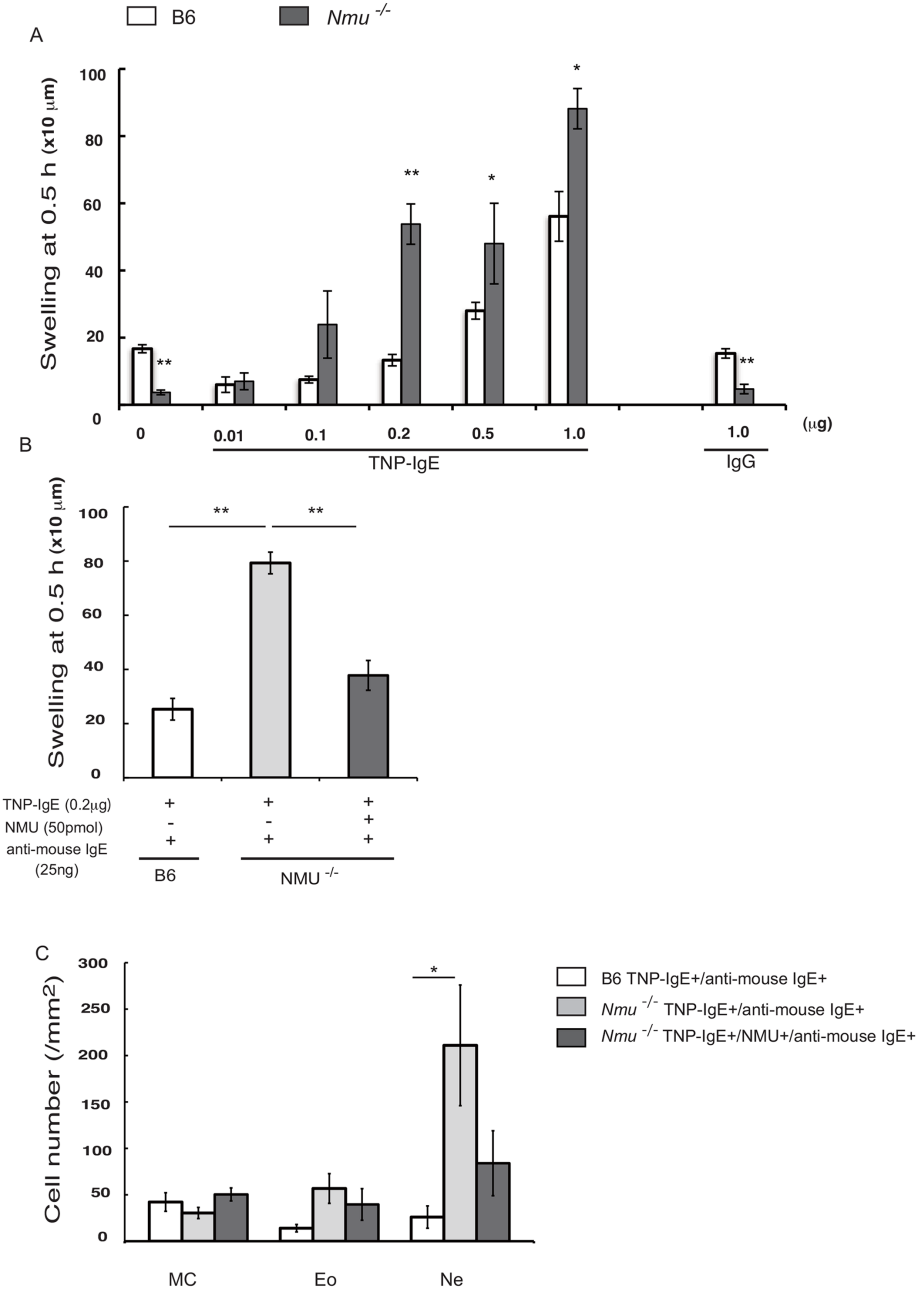
Effects of NMU on ITH mediated by two types of FcεRI cross-linking. (A) ITH responses were induced by TNCB application to the footpad of B6 or *Nmu*^-/-^ mice previously injected with TNP-specific IgE (S1A Fig). Different concentrations of TNP-specific IgE (0.01–1.0 μg in 20 μl of PBS per footpad) were injected 3 days before applying TNCB as follows: 0.05 μg to 0.2 μg of IgE achieved concentrations equivalent to that achieved in the serum of these mice after repeated hapten exposure on days 7–21 (2.5–10 μg/ml). (B) ITH was induced by injecting 0.2 μg of TNP-specific IgE into the footpads of B6 or *Nmu*^-/-^ mice followed by the injection of 25 ng of anti-mouse IgE. NMU (50 pmol) or vehicle (PBS) was injected one day before injection of mice with an anti-mouse IgE (S1B Fig). In B6 mice, ITH was decreased by NMU released from keratinocytes upon injection compared with that of similarly treated *Nmu*^-/-^ mice. (C) Numbers of histologically identifiable dermal mast cells (MC), eosinophils (Eo), and neutrophils (Ne) in the footpads of B6 and *Nmu*^-/-^ mice (n = 4). Data are expressed as the mean ± SEM (n = 10), *P < 0.05, **P < 0.01.

To demonstrate directly the potential role of NMU in preventing IgE-mediated inflammation, we investigated whether ITH mediated by FcεRI cross-linking was enhanced in the absence of NMU. For this purpose, we injected suboptimal to optimal concentrations of TNP-specific IgE (0.01–1.0 μg in 20 μl of PBS per footpad), which corresponded to those present in the sera of *Nmu*^-/-^ mice after repeated hapten exposure on days 7–21 (2.5–10 μg /ml), into the footpads of *Nmu*^-/-^ or B6 mice 3 days before initial application of TNCB (S1A Fig). Application of TNCB only to the footpad caused significant swelling at 0.5 h in B6 but not *Nmu*^-/-^ mice (n = 10, Fig 6A), indicating the proinflammatory effects of endogenous NMU released from keratinocytes in response to TNCB. In B6 mice, ≥0.5μg of TNP-specific IgE was optimal for the development of ITH through hapten-induced FcεRI cross-linking, because significant ITH responses were induced when ≥0.5 μg of TNP-specific IgE was administered to B6 mice. Hapten-specific IgE-mediated ITH was more intense in *Nmu*^-/-^ mice than in B6 mice even on administration of a suboptimal dose of IgE (0.2μg).

To determine whether NMU inhibited the development of ITH induced by another type of FcεRI cross-linking, we injected NMU locally before inducing ITH using an anti-mouse IgE (S1B Fig). As shown in Fig 6B (n = 10), ITH was significantly inhibited. The increase in the number of neutrophils was inhibited by preinjection of NMU (Fig 6C). These results indicate that NMU plays an important role in the down-regulation of ITH mediated by FcεRI cross-linking at suboptimal to optimal levels of IgE.

## Discussion

Because dry skin is a more important cause of scratching than disruption of the cutaneous barrier [2], we predicted that the reduced SSH of *Nmu*^-/-^ mice would render them more susceptible to induction of allergic inflammation than B6 mice. We show here that depletion of NMU from the epidermis contributes to an increase in scratching behavior and subsequent induction of IgE-mediated inflammation associated with reduced SSH. However, depletion of NMU alone is not sufficient to induce IgE-mediated allergic inflammation, because this type of inflammation was not induced in the footpads of *Nmu*^-/-^ mice even after repeated application of hapten (Fig 2D and 2E).

Given the substantial contribution of scratching together with repeated hapten application to the development of IgE-mediated inflammation, it is likely that the significant increase in the number of scratches observed in *Nmu*^-/-^ mice after repeated hapten application is likely a cause, not a consequence, of IgE-mediated allergic inflammation. Our findings therefore indicate that depletion of NMU from the epidermis is associated with reduced SSH. And the depletion of NMU leads to the induction and subsequent acceleration of the onset and severity of IgE-mediated allergic inflammation through increased scratching. Although NMU activates mast cells [13], our results suggest that the presence of NMU in the epidermis serves to mediate the retention of water in the stratum corneum and that the timely release of NMU from the epidermis was induced by exogenous stimuli [13]. Further, NMU attenuates the allergic response induced by the application of an epicutaneous hapten.

Recent studies on filaggrin-deficient flaky tail *(ft)/ft* mice, which serve as a model of AD, demonstrate that filaggrin deficiency alone is insufficient to provoke AD-like inflammation; rather, eliciting this inflammation requires additional factors that reduce barrier competence [29–31]. *Nmu*^-/-^ mice share features with AD patients, as do *ft/ft* mice; for example, *Nmu*^-/-^ mice with an otherwise AD-resistant B6 genetic background have dry skin and develop AD-like skin inflammation characterized by a combined Th1/Th2 cytokine response together with a modest increase in levels of the IL-17-promoting cytokine IL-23, although as for *ft/ft* mice, repeated hapten exposure is required to induce AD-like inflammation. Because IL-4 down-regulates the expression of IL-17 responses [32], a likely explanation for the minimal low levels of Th17 mRNA expression associated with increased IL-23 expression in *Nmu*^-/-^ mice is that the drastic upregulation of IL-4 detected in the skin lesions may have served to reduce the expression of IL-17. Moreover, the frequencies of IL-17-producing NK and γδ^+^ T cells in the draining lymph nodes were higher in *Nmu*^-/-^ than in B6 mice.

An important outcome of the present study is that the footpads of *Nmu*^-/-^ mice, which were not scratched, did not develop IgE-mediated allergic inflammation. These findings indicate that depletion of NMU from the epidermis does not drive IgE-mediated allergic inflammation and that the susceptibility of *Nmu*^-/-^ mice to hapten-induced allergic inflammation depends primarily on scratching a site with low SSH that is repeatedly administered a hapten. We were surprised to find a significant increase in the number of scratches after repeated hapten application to the ears of *Nmu*^-/-^ mice compared with B6 mice, because *Nmu*^-/-^ mice display a slight reduction in spontaneous locomotor activity [33]. Because the inflammatory skin phenotypes associated with increased serum IgE levels were only induced in the ear subjected to scratching, we conclude that dry skin alone is not sufficient to initiate disease and that robust scratching of dry skin is required to initiate and maintain the full inflammatory skin phenotype that shares similarities with AD. Thus, the induction of persistent allergic inflammation requires dry skin associated with the depletion of NMU and is strictly dependent on scratching.

NMU induces certain “proinflammatory” effects upon mast cell activation [13] but can exert beneficial effects that are “anti-inflammatory” under certain inflammatory conditions. It is possible that NMU, which mediates certain “proinflammatory” effects of acute inflammation, limits the duration or intensity of chronic allergic inflammation as well. When released in the skin at the appropriate time, NMU may play an important physiological role in the down-regulation of IgE-mediated allergic inflammation. Our present data, which support this possibility, show that IgE-mediated allergic inflammation as manifested by FcεRI cross-linking is much more easily induced using suboptimal doses of IgE in the absence of NMU in the epidermis (Fig 6A).

In conclusion, our results provide evidence for a dual, stage-dependent role of NMU in hapten-induced inflammation. NMU serves as a principal mediator of innate-type mast cell activation at an early stage [13]; here, we show that NMU is a negative regulator of IgE-mediated allergic inflammation at a later stage. NMU would be required to maintain epidermal homeostasis in the setting of excessive proinflammatory responses, such as repeated hapten application. Moreover, NMU may act as a critical factor required for the initiation of effective innate immune responses while limiting self-induced tissue damage. The actions of NMU on allergic inflammation are likely complex. For example, NMU can maintain SSH through its potential capacity to retain water in the skin and thereby prevent itching. Because it is virtually impossible to avoid exposure to various allergens and pathogens, therapeutic targeting of NMU might serve to better control IgE-mediated allergic inflammation in atopic individuals. In view of the possibility that NMU acts locally in response to a scratching-induced insult, it may represent a central target for the treatment of pruritus.

## Supporting Information

S1 Fig Experimental design for investigating the effects of NMU on ITH responses mediated by two types of FcεRI cross-linking. In the first type (A), FcεRI cross-linking was induced by TNCB application to footpads previously sensitized with TNP-IgE. In the second type (B), FcεRI cross-linking was induced by injecting anti-IgE into footpads previously sensitized with TNP-IgE.
(TIF)

## Abbreviations

AD: atopic dermatitis
ITH: immediate-type hypersensitivity
NMU: neuromedin U
SSH: skin surface hydration
TEWL: transepidermal water loss
TNCB: 2, 4, 6-trinitro-1-chlorobenzene
