# Supplementary figures and images for: Epidermal Neuromedin U Attenuates IgE-Mediated Allergic Skin Inflammation
Source: PLoS One. 2016 Jul 27;11(7):e0160122. doi: 10.1371/journal.pone.0160122 (PMC4963095; doi:10.1371/journal.pone.0160122)

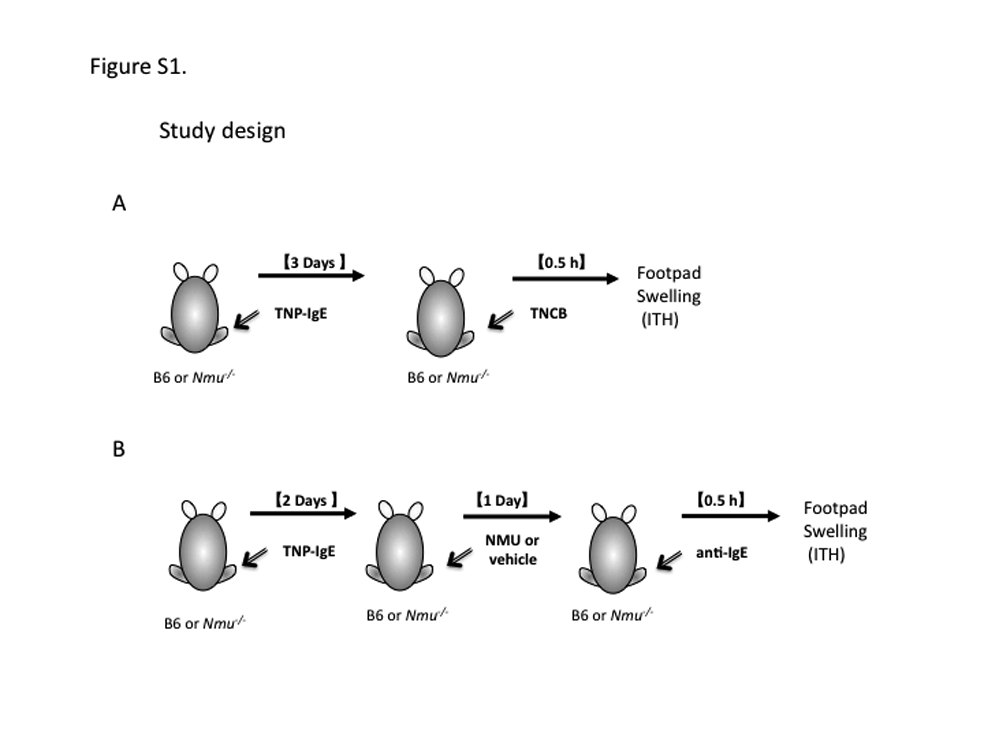

Supplement: S1 Fig — In the first type (A), FcεRI cross-linking was induced by TNCB application to footpads previously sensitized with TNP-IgE. In the second type (B), FcεRI cross-linking was induced by injecting anti-IgE into footpads previously sensitized with TNP-IgE. (TIF) [file pone.0160122.s001.tif]
